# *ESR1* gene promoter region methylation in free circulating DNA and its correlation with estrogen receptor protein expression in tumor tissue in breast cancer patients

**DOI:** 10.1186/1471-2407-14-59

**Published:** 2014-02-04

**Authors:** Joaquina Martínez-Galán, Blanca Torres-Torres, María Isabel Núñez, Jesús López-Peñalver, Rosario Del Moral, José Mariano Ruiz De Almodóvar, Salomón Menjón, Ángel Concha, Clara Chamorro, Sandra Ríos, Juan Ramón Delgado

**Affiliations:** 1 Department of Medical Oncology, Hospital Universitario Virgen de las Nieves, University of Granada, Avenida de las Fuerzas Armadas s/n, 18011 Granada, Spain; 2 Centro de Investigación Biomédica, Universidad de Granada, Granada, Spain; 3 Departamento de Radiología y Medicina Física, Facultad de Medicina de Granada, Granada, Spain; 4 Servicio de Oncología Radioterápica, Hospital Universitario Virgen de las Nieves, Granada, Spain; 5 Servicio de Ginecología, Hospital Universitario Virgen de las Nieves, Granada, Spain; 6 Servicio de Anatomía Patológica, Hospital Universitario Virgen de las Nieves, Granada, Spain

**Keywords:** Breast cancer, Methylation, Luminal phenotypes

## Abstract

**Background:**

Tumor expression of estrogen receptor (ER) is an important marker of prognosis, and is predictive of response to endocrine therapy in breast cancer. Several studies have observed that epigenetic events, such methylation of cytosines and deacetylation of histones, are involved in the complex mechanisms that regulate promoter transcription. However, the exact interplay of these factors in transcription activity is not well understood. In this study, we explored the relationship between ER expression status in tumor tissue samples and the methylation of the 5′ CpG promoter region of the estrogen receptor gene (*ESR1*) isolated from free circulating DNA (fcDNA) in plasma samples from breast cancer patients.

**Methods:**

Patients (n = 110) with non-metastatic breast cancer had analyses performed of ER expression (luminal phenotype in tumor tissue, by immunohistochemistry method), and the *ESR1*-DNA methylation status (fcDNA in plasma, by quantitative methylation specific PCR technique).

**Results:**

Our results showed a significant association between presence of methylated *ESR1* in patients with breast cancer and ER negative status in the tumor tissue (p = 0.0179). There was a trend towards a higher probability of *ESR1*-methylation in those phenotypes with poor prognosis i.e. 80% of triple negative patients, 60% of HER2 patients, compared to 28% and 5.9% of patients with better prognosis such as luminal A and luminal B, respectively.

**Conclusion:**

*S*ilencing, by methylation, of the promoter region of the *ESR1* affects the expression of the estrogen receptor protein in tumors of breast cancer patients; high methylation of *ESR1*-DNA is associated with estrogen receptor negative status which, in turn, may be implicated in the patient’s resistance to hormonal treatment in breast cancer. As such, epigenetic markers in plasma may be of interest as new targets for anticancer therapy, especially with respect to endocrine treatment.

## Background

The therapeutic options indicated for patients with breast cancer continue to be based, principally, on clinical-pathology criteria. The incorporation of new immunohistochemistry and molecular biology markers into the diagnosis has advanced the knowledge of potential markers of prognosis and prediction of response to endocrine therapy in breast cancer. One of the biomarkers most used is the expression of estrogen and of progesterone receptors (ER and PR, respectively) [1]. However, only 2/3 of the patients diagnosed with breast cancer express ER at diagnosis (ER+), while the other 1/3 of the cases do not express the receptors (ER-), and which is associated with non-differentiated tumors, with high cell proliferation index, poor response to endocrine therapy and poor prognosis [2]. Some tumors which are ER + at the time of diagnosis become ER- in the course of the clinical evolution of the disease [3]. Also, 30-40% of the ER + patients will develop resistance to the anti-estrogen treatment and which will favor the appearance of distant metastases, and death.

To date, the molecular bases of the response to endocrine therapy are poorly understood. Recent studies have shown that heterogeneity of response and prediction of response to chemotherapy and sensitivity to hormone therapy is based on “molecular portraits” [4,5]. However, the list of genes implicated in prognosis may or may not necessarily relate to the clinical results obtained in response to treatment [6] and, as such, warrants further investigation.

To date, methylation of DNA is known as an epigenetic phenomenon which plays a decisive role in the regulation of signal translation processes. Under physiological conditions, this epigenetic event influences which genes are activated during the process of normal cell differentiation [7], the maintenance of “genetic imprinting” [8], inactivation of the X chromosome [9], genetic transcription repression [10] and the suppression of regions of parasite DNA [11]. However, these epigenetic events, when aberrant, have a determining role in the development of malignant tumor processes [12,13] and, as well, the suggestion is of an involvement in resistance to chemotherapy, radiotherapy and hormone therapy [14].

ER role is key since up to 1/3 of the patients who do not express ER (i.e. ER-), rarely respond to hormone treatment [15]. ER is coded-for by the *ESR1* gene located at chromosome 6q25.1, the promoter region of which contains a linked CpG sequence in exon 1. In breast cancer cell lines such as MCF-7, T47-d and ZR75-1 [16] that express ER (i.e. ER+) this region is observed to be non-methylated, and is similar to that occurring in normal tissue. However, in cells lines from ER- breast cancer, such as MDA-MB-231, MDA-MB-435, MDA-MB-468, Hs578t and MCF-7/Adr, methylation is observed in >50% of cases [17]. Hence, determining the methylation status of the promoter region of the *ERS1* could be critical since it represents one of the mechanisms by which the loss of ER expression is associated with breast cancer diagnosis. Blocking this process of methylation could be important since this could lead to patients who are resistant to hormone treatment becoming sensitive to hormone treatment [18].

Based on the literature, as well as on the experience in our own research group [19-21], we designed the present study to assess whether ER- expression in tumor tissue correlates with methylated status of the *ESR1* in serum i.e. a mechanism of gene silencing that can explain, at least in part, the lack of hormone therapy efficacy in breast cancer. Also, we sought insight into fcDNA methylation and tumor phenotypes: Luminal A (LA), Luminal B (LB), Triple negative (TN) and Her2 +.

## Methods

### Study population

A total of 110 patients diagnosed as having non-metastatic breast cancer in the *Hospital Universitario Virgen de las Nieves de Granada* (Spain) were included in the study. Patient characteristics are summarized in Table 1. The study was approved by the Institutional Ethics Committee of the *Hospital Universitario Virgen de las Nieves de Granada*, and written informed consent was obtained from all study participants.

**Table 1:** Demographic characteristics of the breast cancer patients

**Characteristic**		**Cases**	**SD**
		**N = 110**	
Mean age; years (range)		58 (32–88)	12.4
Mean age at menarche; years (range)		13 (10–17)	1.4
Mean age at menopause; years (range)		49 (39–59)	3.8
Menopausal status			
	Pre-menopause	30.8%	
	Post-menopause	69.2%	
Mean age at first live birth; years (range)		25 (18–41)	3.9
Mean age at last live birth; years (range)		32 (20–42)	5.2
Breastfeeding			
	Yes	82 (76.6%)	
	No	23 (21.5%)	
Breastfeeding; months (range)		6 (1–36)	5.1

SD: standard deviation.

### Collection and processing of samples and DNA preparation

Blood samples (10 ml) were taken by venipuncture from all the study patients on introduction into the study and before the administration of any medication. The blood samples were collected into EDTA Vacutainer® tubes and coded before processing to ensure blinding with respect to sample provenance. The samples were transported at room temperature to the laboratory, centrifuged at 2000 g for 10 min at room temperature, the plasma obtained was distributed in 1 ml aliquots into 1 ml criotubes, and stored at -80°C until needed for processing.

- DNA isolation: DNA from plasma samples (2 ml per column) was obtained using QIAmp DNA Blood kit (QIAGEN Inc., CA) according to manufacturer’s recommendations. A final elution volume was 200 μl and the extracted DNA was quantified spectrophotometrically. The amount of DNA recovered, measured as μg/sample, was 0.431 ± 0.019 (mean value ± standard error of the mean). The fcDNA samples were stored at −80°C until needed for analysis.

### Quantitative Methylation Specific (QMS) polymerase chain reaction (PCR) analysis

- DNA bisulfite modification: Identical DNA sequences that differ only in methylation status can be amplified by means of Quantitative Methylation Specific PCR (QMS-PCR) [22]. Reagents required for the bisulfite modification of fcDNA were supplied in the CpGenome™ DNA Modification Kit (Intergen, MA). The process was performed according to manufacturer’s recommendations. Sufficient fcDNA can be recovered to perform QMS-PCR from an amount of starting material as small as 0.001 μg. In brief, 100 μl of extracted fcDNA was treated with sodium bisulfite for 16 h, thereby converting all unmethylated cytosines to uracils, but leaving methylcytosines unaltered. After purification, the fcDNA obtained was dissolved in 20 μl of TE buffer and the modified DNA was spectrophotometrically quantified. Efficiency of fcDNA recovery after bisulfite modification was around 55% (data not shown). Recovered bisulfite-treated fcDNA (1 μl) was used in each well for SYBR green reaction. Modified DNA of standards and samples are stable for at least 2 months at −80°C. A sample of bisulfite-modified universally-methylated genomic DNA, (CpGenomeTM Universal Methylated DNA, Intergen, New York, NY) treated in the same way as patient samples and the concentration adjusted, after modification, to 2 μg/ml (quantified spectrophotometrically), served as internal standard in preparing serial dilutions (from 1 to 1/128 with MiliQ water) to construct a standard curve for Real-Time QMS-PCR. Each multi-well plate contained patient samples, serial dilutions of completely methylated DNA for constructing calibration curves, positive controls, and two wells with water used as negative controls (“blanks”). In all experiments, correlation coefficients for the calibration curves were >0.98, slopes ranged from 3.2 to 3.4, and PCR efficiencies were around 100%.

As found by other authors [19,20,23,24], some gene promoters were frequently observed to have methylated DNA in the plasma of cancer patients, albeit traces of methylated DNA may also be found in plasma of patients without cancer when highly sensitive quantitative techniques are used. Hence, cut-off points for the *ESR1* methylated promoter was established from the receiver operating characteristics (ROC) curves i.e. selecting values that gave the maximal likelihood ratio (in current case the cut-of value was 0.02 relative units) [20]. Assuming levels of methylation of *ESR1* < 0.02 relative units, “test of methylation (−)”, was indicative of absence of the disease (physiological) while levels of methylation of *ESR1* > 0.02 relative units measured in the plasma “test of methylation (+)” was indicative of presence of breast cancer (pathological level of methylation). Once the distribution of cases was established in the two groups as “test of methylation (−)” and “test of methylation (+)”, the study proceeded to assess whether this characteristic was associated with the phenotype ER(+) and ER(−) in tumor tissue.

Using a method developed previously in our group [20,21], Quantitative Methylation Specific PCR (QMS-PCR) was performed with the iQ SYBR-Green Supermix Kit (BioRad Laboratories; Hercules, CA) according to the manufacturer’s protocol. The sequences of the primers for *ESR1* were selected from previous publications: *ESR1* Genebank 2099 location to transcription start Promoter A [25]. The fluorescence value corresponding to each sample was converted into relative units of universally methylated DNA (umDNA; μg/mL) using the corresponding calibration curve adjusted by the software program of the QMS-PCR equipment. The PCR reaction was conducted in 96-well plates which contained: patient samples, successive dilutions for the calibration curve, 2 positive controls, and 2 negative controls or “blanks” (without DNA). In all cases the correlation coefficients for the calibration curves were ≥ 0.98, the linear slope was between 3.02 and 3.2, and the PCR efficacy was between 85 and 110%.

### Immunohistochemical staining for ER, PR and HER2 expression in tumor tissue

Starting with surgically excised tissue preserved in formol, tumor pieces were embedded in paraffin and processed for staining with eosin-hematoxylin. ER and PR expression were evaluated in tumor tissue using the DAKO HORIZON automatic processor (Techmate Horizon). Monoclonal antibody kits were purchased from the manufacturer (DAKO M 7047 Clone 185 for ER and DAKO M 3569 Clone 636 for PR) and used according to the manufacturer’s instructions. Nuclear staining indicates positive or negative. Positivity is expressed as intensity of staining and graded as weak (+) moderate (++) and strong (+++), and as percentage of cells stained. Subsequently, HER2 amplification in the tumor sample was with the DAKO K5206 kit. In those cases with HER2 (++), further analysis was with FISH using the DAKO K 5331, HER FISH PharmDxTM kit and hybridized in the DAKO HYBRIDIZER.

### Statistical methods

The data obtained were analyzed using the following statistical tests: 1) description of the demographic and clinical-pathology variables using means, medians, percentiles, ranges and standard deviations; 2) relationships between ER expression in tumor and quantitative specimen level of *ESR1* methylation using Chi-square (and Fisher’s exact) test.

## Results and discussion

The demographic and clinical-pathology characteristics of the participants at study entry are summarized in Tables 1 and 2. The cut-off points for *ESR1* methylated promoter in plasma samples were established from the ROC curves, selecting values that gave the maximal likelihood ratio of 0.02 relative units for *ESR1.* From our results previously obtained [20], we assume that levels of methylation of *ESR1* >0.02 relative units are indicative of the presence of disease (test “+” indicating breast cancer and pathologic level of methylation *ESR1*) whereas level of methylation <0.02 relative units indicating absence of disease (test “–” indicating no presence of breast cancer and physiologic level of methylation *ESR1*). Subsequently we sought correlations between methylation levels of the *ESR1* promoter in the fcDNA samples and the absence of transcription and, in turn, with the lack of ER expression in tumor tissue.

**Table 2:** Clinico-pathological characteristics of the breast cancer patients

**Characteristic**		**Cases**	**% of total**
		**N = 110**	
Histological type			
	Invasive ductal carcinoma	82	74.5
	Invasive lobulillar carcinoma	10	9
	Invasive mixed carcinoma	7	6.3
	Others	11	10
Histological grade			
	Grade I	20	18.7
	Grade II	40	37.4
	Grade III	35	32.7
	Unknown	12	11.2
Pathological T			
	T1	64	58
	T2	43	39
	T3	3	2.7
Pathological N			
	N0	75	68
	N1	35	32
	N2	0	0
Luminal phenotype			
	Luminal A	39	36.4
	Luminal B	22	20.5
	Triple Negative	15	14
	Her2-neu	10	9.3
	Unknown	21	19.6

Using Chi-square (and Fisher’s exact) test we studied the relationship between a positive test value for *ESR1-*DNA promoter methylation in plasma sample and ER- status in excised tumor samples from patients with breast cancer (p < 0.05). Similarly, we checked for correlations between negative test value for *ESR1 vs.* ER + in tumor tissue and observed that the relationship was statistically significant (p < 0.05). Results obtained showed an association between presence of methylated *ESR1* in fcDNA from patients with breast cancer and ER- status in tumor, an observation that would be expected if promoter methylation leads to silencing of gene expression, and *vice versa*.

**Figure 1:**
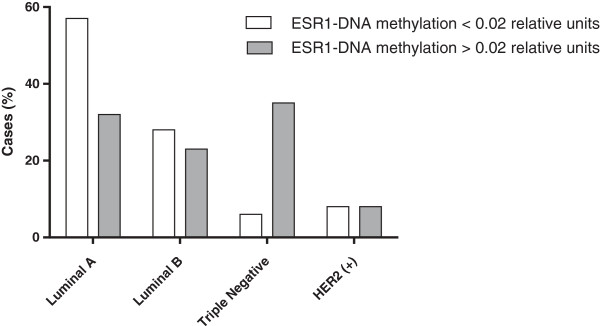
Histogram showing breast cancer subtype of poorer prognosis (TN and Her2) have higher percentage of ESR1-DNA promoter methylation > 0.02 relative units, while those phenotypes with better prognosis (luminal A and luminal B) the percentage of ESR1-DNA promoter methylation is < 0.02 relative units.

Similarly, we investigated the relationship between the gene expression silencing mechanism (using methylation of the *ESR1* promoter i.e. epigenetic silencing) and the luminal phenotype of the tumor tissue; the hypothesis being that the predominant role of methylation is gene silencing (i.e. restricted expression) in the tumor. The Chi-square (and Fisher’s exact) test showed a significantly (p < 0.05) higher percentage of *ESR1* promoter methylation in those phenotypes with poorer prognosis i.e. 80% of the triple negative patients and 60% of patients with HER2 compared to 28% and 36% of patients with phenotypes of better prognosis such as luminal A and luminal B, respectively (Table 3, Figure 1).

**Table 3:** **Percentage *ER *hypermethylation in relation to tumor phenotype**

***Test result***	**Luminal A**		**Luminal B**		**Triple negative**		**HER2(+)**		**P**
	**n**	**%**	**n**	**%**	**n**	**%**	**n**	**%**	
*(−) ESR1 < 0.02*	28	(71%)	14	(64%)	3	(20%)	4	(40%)	<0.05
*(+) ERS1 > 0.02*	11	(28%)	8	(36%)	12	(80%)	6	(60%)	<0.05

Based on these findings, we analyzed whether there could be subgroups of patients within each luminal phenotype such that a methylation profile could explain, at least in part, why some cases develop distant metastases despite having a good prognostic phenotype. We observed that, within each subgroup segregated with respect to *ESR1* methylation in the fcDNA sample, there was a tendency towards a lower survival at 4.5 years of follow-up in each phenotype that had methylated *ESR1* (*ESR1* methylation level >0.02) compared to the subgroup in which the *ESR1* methylation level was <0.02. However, these differences were not statistically significant, perhaps because of the small number of cases (Table 4).

**Table 4:** Differences in presentation of aberrant ER methylation within the luminal phenotype subgroups

**Phenotype**	**N**	**OS at 5 years**	**P**
*ER* non-methylated			
Luminal A	28	93%	NS
Luminal B	14	92%	NS
Triple Negative	3	80%	NS
Her2	4	75%	NS
*ER* methylated			
Luminal A	11	82%	NS
Luminal B	8	86%	NS
Triple negative	12	75%	NS
Her2	6	67%	NS

OS: overall survival.

It has been well documented that the ER expression in tumor tissue of breast cancer represents one of the principal prognostic factors of long-term survival, and is predictive of the disease response to hormone therapy. As such, the patients with tumors that are ER + are associated with a more favorable prognosis and the hormonal treatment of which is based on tamoxifen and/or aromatase inhibitors. However, in those patients that present tumors with ER-, the hormone treatment has little or no therapeutic value but there is an element of impact on the prognosis of the disease i.e. ER- is associated more frequently with those cases in which the course of the disease is more adverse. Around 25% of breast cancer patients do not express ER at the time of diagnosis and, as such, are resistant to hormonal therapy [2]. Also, in some cases the initial expression of ER + can change to ER- and negatively impact on the long-term course of the disease due to loss of sensitivity to the hormonal treatment [26]. Further research is warranted to explain this finding of change in phenotype [27].

In our literature search, we did find a few studies that correlated the epigenetic profile of methylation and its relationship with ER expression status but no study correlating the methylation with luminal phenotype. There have been earlier studies that investigated the role of methylation of various genes in search of independent markers of prognosis in breast cancer [28]. However, methylation of the promoter region of the *ESR1* gene has received little attention in relation to the absence of ER expression in the corresponding tumor. Authors such as Widschwendter *et al.*[29] described a significant relationship (p = 0.015) between the *APC* methylation status and hormonal receptor status predictors in patients with breast cancer. Others studies, such as that by Yang *et al.*[30], suggested that, in ER + patients, there may be a higher frequency of hypermethylation of the promoter of the *Twist* gene, while methylation of the *CDH1* gene occurs with higher frequency in patients the ER- tumors. Recent articles suggest that the hypermethylation of this gene promoter occurs predominantly in triple negative breast cancer [31]. This hypothesis is contested by other authors [32].

In the present study of cases in which we observed hypermethylation of the *ESR1* promoter in plasma of the patients with breast cancer, we undertook an analysis of epigenetic signal silencing of the *ESR1* gene in patients segregated with respect to luminal phenotypes, to the histopathology of the tumor, and to ER protein expression in the tumor. We evaluated *ESR1*-fcDNA methylation in peripheral plasma in relation to ER expression in tumor tissues. The results showed that the methylation of *ERS*1-fcDNA was correlated significantly with the absence of ER protein expression in the tumor, and *vice versa* (p = 0.018). This result is in accordance with that described by Lapidus *et al.* and Ottaviano *et al.*[18,33] in breast cancer cell lines. Essentially, these authors described that the promoter located in exon 1 of the *ESR1* gene is observed to be highly methylated in the cell lines that do not express ER protein and, conversely, is not methylated in normal breast tissue as well as in the cell lines of breast cancer that express functioning estrogen receptors.

With respect to the luminal phenotype and *ESR1* methylation status, we observed that, in those cases with the better prognosis phenotypes (luminal A and luminal B) the predominant result of the evaluation of *ESR1-*fcDNA methylation was negative i.e. there was a correlation between ER expression and better prognosis while, in the phenotype with poor prognosis (Her2-neu and triple negative), the evaluation was predominantly positive (p < 0.05). Further analysis of each luminal phenotype with respect to methylated *ESR1* >0.02 relative units *versus* methylated *ESR1* <0.02 relative units, showed that, within each subgroup, those that presented methylated *ESR1 *(and as such not expressing ER receptors) had a tendency towards shorter overall survival than those with a methylated *ESR* level *<* 0.02 relative units*.* Hence, it appears that the molecular variant of the methylated promoter region of the *ESR1* gene carries prognostic information that is additional to the prognostic factors that are currently used in the breast cancer clinic.

From the above observations, the most relevant of the results obtained is that methylation, as an epigenetic event, can be one of the mechanisms implicated in the non-expression of ER in the tumor. We observed significant correlation between *ESR1* DNA methylation in peripheral blood and silencing of ER expression in the tumor, and *vice versa*. This finding is of considerable interest especially since we observed, as well, the correlation between *ESR1*-fcDNA methylation and the ER expression in relation to tumor phenotype. The greater expression of the estrogen receptors in the phenotypes that are known to have a better prognosis (LA and LB) while showing significantly lower levels of *ESR1* in peripheral blood. This contrasts with those phenotypes in which the ER expression is low or not present which, in turn is associated with poorer prognosis (triple negative and Her2). The mechanisms involved in the absence of ER expression are not known, and neither are the reasons for non-response to hormonal treatment. Of note, as well, is that tumors that express ER at the time of diagnosis can cease expressing ER over the time-course of the disease. Perhaps one of the causal events in the absence of ER expression (and its negative impact on the clinical evolution of the disease) is that tumors expressing ER at the time of diagnosis are methylated. In studies conducted to date, the known genetic alterations do not fully explain the processes but, based on the results from our study, perhaps methylation is the key to explaining, at least in part, some of the observations. Also, given that methylation is a reversible event, analyzing this reversibility can be useful in identifying the crucial point in the success, or otherwise, of hormonal treatment of breast cancer.

## Conclusions

Our results indicate that the silencing of gene expression by methylation of the promoter region of the *ESR1* gene of the estrogen receptor has an important role in the expression of the protein for which this gene codes (i.e. the estrogen receptor) in the primary tumor. The methylation of ESR1 in peripheral bold correlates significantly with the non-expression of ER in excised tumor tissue. As such, this measurement may add prognostic value in identifying luminal phenotypes with poor prognosis and, as well, those with potentially greater resistance to hormonal treatment. The protein is absent in ER- tumors when methylation occurs in the *ESR1* promoter, and *vice versa*. These data demonstrate that analysis of methylation in the DNA from peripheral circulation (fcDNA) can help in determining prognosis or in predicting response to certain types of treatment. Of more importance, perhaps, is that since methylation is a reversible event, its modulation can constitute a future therapeutic target.

Reversal of methylation status and, as such, the genetic expression resulting from this mechanism, could cause a recovery of function of those genes responsible for the regulation of the normal cell cycle which, as a result of an alteration in the methylation pattern, are abnormally silenced. This could be of considerable interest because such an easily measured analyte (*ESR1* DNA methylation in peripheral blood) can serve as biomarker, and probable therapeutic target against breast cancer.

## Abbreviations

ESR1: Estrogen receptor1 gene; 14-3-3-σ: Stratifin; ER: Estrogen receptor; fcDNA: Free circulating DNA; PR: Progesterone receptor; LA: Luminal A; LB: Luminal B; TN: Triple negative; QMS - PCR: Quantitative methylation-specific-polymerase chain reaction; ROC curve: Receiver operating characteristic curve.

## Competing interests

The authors declare that they have no competing interests.

## Authors’ contributions

JM-G, SM, JRD and RdelM were significantly involved in patient recruitment and management, and placing the experimental findings into clinical perspective. BT-T, MIN, AC, CC, JL-P and SR conducted the *in vitro* analyses. JM-G and JM-A were involved in the interpretation and discussion of results; JM-G, JMRdeA and JRD conceived and designed the study, interpreted the data, and revised the manuscript. All the authors have read and approved the final manuscript.

## Pre-publication history

The pre-publication history for this paper can be accessed here:

http://www.biomedcentral.com/1471-2407/14/59/prepub

## References

[B1] Sotiriou C Desmedt C Gene expression profiling in breast cancer Ann Oncol 2006 17 Suppl 10 x259 x262 10.1093/annonc/mdl270 17018735

[B2] Hortobagyi GN Treatment of breast cancer N Engl J Med 1998 339 14 974 984 10.1056/NEJM199810013391407 9753714

[B3] Yang X Yan L Davidson NE DNA methylation in breast cancer Endocr Relat Cancer 2001 8 2 115 127 10.1677/erc.0.0080115 11446343

[B4] Sotiriou C Neo SY McShane LM Korn EL Long PM Jazaeri A Martiat P Fox SB Harris AL Liu ET Breast cancer classification and prognosis based on gene expression profiles from a population-based study Proc Natl Acad Sci USA 2003 100 18 10393 10398 10.1073/pnas.1732912100 12917485 PMC193572

[B5] Michiels S Koscielny S Hill C Prediction of cancer outcome with microarrays: a multiple random validation strategy Lancet 2005 365 9458 488 492 10.1016/S0140-6736(05)17866-0 15705458

[B6] Ein-Dor L Kela I Getz G Givol D Domany E Outcome signature genes in breast cancer: is there a unique set? Bioinformatics 2005 21 2 171 178 10.1093/bioinformatics/bth469 15308542

[B7] Li E Beard C Forster AC Bestor TH Jaenisch R DNA methylation, genomic imprinting, and mammalian development Cold Spring Harb Symp Quant Biol 1993 58 297 305 10.1101/SQB.1993.058.01.035 7956042

[B8] Forne T Oswald J Dean W Saam JR Bailleul B Dandolo L Tilghman SM Walter J Reik W Loss of the maternal H19 gene induces changes in Igf2 methylation in both cis and trans Proc Natl Acad Sci USA 1997 94 19 10243 10248 10.1073/pnas.94.19.10243 9294195 PMC23347

[B9] Heard E Avner P Role play in X-inactivation Hum Mol Genet 1994 3 Spec No 1481 1485 7849742 10.1093/hmg/3.suppl_1.1481

[B10] Chan MF Liang G Jones PA Relationship between transcription and DNA methylation Curr Top Microbiol Immunol 2000 249 75 86 10802939 10.1007/978-3-642-59696-4_5

[B11] Yoder JA Walsh CP Bestor TH Cytosine methylation and the ecology of intragenomic parasites Trends Genet 1997 13 8 335 340 10.1016/S0168-9525(97)01181-5 9260521

[B12] Paulsen M Ferguson-Smith AC DNA methylation in genomic imprinting, development, and disease J Pathol 2001 195 1 97 110 10.1002/path.890 11568896

[B13] Dumitrescu RG Epigenetic markers of early tumor development Methods Mol Biol 2012 863 3 14 10.1007/978-1-61779-612-8_1 22359284

[B14] Kastl L Brown I Schofield AC Altered DNA methylation is associated with docetaxel resistance in human breast cancer cells Int J Oncol 2010 36 5 1235 1241 20372798 10.3892/ijo_00000607

[B15] Lapidus RG Nass SJ Davidson NE The loss of estrogen and progesterone receptor gene expression in human breast cancer J Mammary Gland Biol Neoplasia 1998 3 1 85 94 10.1023/A:1018778403001 10819507

[B16] Ray S Fry MJ Darbre PD Enhanced sensitivity to rapamycin following long-term oestrogen deprivation in MCF-7, T-47-D and ZR-75-1 human breast cancer cells J Endocrinol 2011 208 1 21 29 10.1677/JOE-10-0137 20947540

[B17] Lapidus RG Nass SJ Butash KA Parl FF Weitzman SA Graff JG Herman JG Davidson NE Mapping of ER gene CpG island methylation-specific polymerase chain reaction Cancer Res 1998 58 12 2515 2519 9635570

[B18] Ottaviano YL Issa JP Parl FF Smith HS Baylin SB Davidson NE Methylation of the estrogen receptor gene CpG island marks loss of estrogen receptor expression in human breast cancer cells Cancer Res 1994 54 10 2552 2555 8168078

[B19] Valenzuela MT Galisteo R Zuluaga A Villalobos M Nunez MI Oliver FJ Ruiz de Almodovar JM Assessing the use of p16(INK4a) promoter gene methylation in serum for detection of bladder cancer Eur Urol 2002 42 6 622 628 discussion 628–630 10.1016/S0302-2838(02)00468-2 12477660

[B20] Martinez-Galan J Torres B Del Moral R Munoz-Gamez JA Martin-Oliva D Villalobos M Nunez MI Luna Jde D Oliver FJ Ruiz de Almodovar JM Quantitative detection of methylated ESR1 and 14-3-3-sigma gene promoters in serum as candidate biomarkers for diagnosis of breast cancer and evaluation of treatment efficacy Cancer Biol Ther 2008 7 6 958 965 10.4161/cbt.7.6.5966 18379196

[B21] Zurita M Lara PC del Moral R Torres B Linares-Fernandez JL Arrabal SR Martinez-Galan J Oliver FJ Ruiz de Almodovar JM Hypermethylated 14-3-3-sigma and ESR1 gene promoters in serum as candidate biomarkers for the diagnosis and treatment efficacy of breast cancer metastasis BMC Cancer 2010 10 217 10.1186/1471-2407-10-217 20487521 PMC2889892

[B22] Herman JG Graff JR Myohanen S Nelkin BD Baylin SB Methylation-specific PCR: a novel PCR assay for methylation status of CpG islands Proc Natl Acad Sci USA 1996 93 18 9821 9826 10.1073/pnas.93.18.9821 8790415 PMC38513

[B23] Hoque MO Topaloglu O Begum S Henrique R Rosenbaum E Van Criekinge W Westra WH Sidransky D Quantitative methylation-specific polymerase chain reaction gene patterns in urine sediment distinguish prostate cancer patients from control subjects J Clin Oncol 2005 23 27 6569 6575 10.1200/JCO.2005.07.009 16170165

[B24] Bastian PJ Palapattu GS Lin X Yegnasubramanian S Mangold LA Trock B Eisenberger MA Partin AW Nelson WG Preoperative serum DNA GSTP1 CpG island hypermethylation and the risk of early prostate-specific antigen recurrence following radical prostatectomy Clin Cancer Res 2005 11 11 4037 4043 10.1158/1078-0432.CCR-04-2446 15930338

[B25] Sasaki M Tanaka Y Perinchery G Dharia A Kotcherguina I Fujimoto S Dahiya R Methylation and inactivation of estrogen, progesterone, and androgen receptors in prostate cancer J Natl Cancer Inst 2002 94 5 384 390 10.1093/jnci/94.5.384 11880477

[B26] Kuukasjarvi T Kononen J Helin H Holli K Isola J Loss of estrogen receptor in recurrent breast cancer is associated with poor response to endocrine therapy J Clin Oncol 1996 14 9 2584 2589 8823339 10.1200/JCO.1996.14.9.2584

[B27] Fuqua SA Allred DC Elledge RM Krieg SL Benedix MG Nawaz Z O’Malley BW Greene GL McGuire WL The ER-positive/PgR-negative breast cancer phenotype is not associated with mutations within the DNA binding domain Breast Cancer Res Treat 1993 26 2 191 202 10.1007/BF00689692 8219256

[B28] Muller HM Widschwendter A Fiegl H Ivarsson L Goebel G Perkmann E Marth C Widschwendter M DNA methylation in serum of breast cancer patients: an independent prognostic marker Cancer Res 2003 63 22 7641 7645 14633683

[B29] Widschwendter M Siegmund KD Muller HM Fiegl H Marth C Muller-Holzner E Jones PA Laird PW Association of breast cancer DNA methylation profiles with hormone receptor status and response to tamoxifen Cancer Res 2004 64 11 3807 3813 10.1158/0008-5472.CAN-03-3852 15172987

[B30] Yang J Mani SA Donaher JL Ramaswamy S Itzykson RA Come C Savagner P Gitelman I Richardson A Weinberg RA Twist, a master regulator of morphogenesis, plays an essential role in tumor metastasis Cell 2004 117 7 927 939 10.1016/j.cell.2004.06.006 15210113

[B31] Prabhu JS Wahi K Korlimarla A Correa M Manjunath S Raman N Srinath BS Sridhar TS The epigenetic silencing of the estrogen receptor (ER) by hypermethylation of the ESR1 promoter is seen predominantly in triple-negative breast cancers in Indian women Tumour Biol 2012 33 2 315 323 10.1007/s13277-012-0343-1 22362381

[B32] Ramezani F Salami S Omrani MD Maleki D CpG island methylation profile of estrogen receptor alpha in Iranian females with triple negative or non-triple negative breast cancer: new marker of poor prognosis Asian Pac J Cancer Prev 2012 13 2 451 457 10.7314/APJCP.2012.13.2.451 22524805

[B33] Lapidus RG Ferguson AT Ottaviano YL Parl FF Smith HS Weitzman SA Baylin SB Issa JP Davidson NE Methylation of estrogen and progesterone receptor gene 5′ CpG islands correlates with lack of estrogen and progesterone receptor gene expression in breast tumors Clin Cancer Res 1996 2 5 805 810 9816234

